# Sublethal effects of imidacloprid on the performance of the bird cherry-oat aphid *Rhopalosiphum padi*

**DOI:** 10.1371/journal.pone.0204097

**Published:** 2018-09-20

**Authors:** Wenqiang Li, Zengbin Lu, Lili Li, Yi Yu, Song Dong, Xingyuan Men, Baohua Ye

**Affiliations:** 1 Institute of Plant Protection, Shandong Academy of Agricultural Sciences, Jinan, China; 2 College of Plant Protection, Shandong Agricultural University, Tai’an, China; 3 Maize Research Institute, Shandong Academy of Agricultural Sciences, National Engineering Laboratory of Wheat and Maize, Key Laboratory of Biology and Genetic Improvement of Maize in Northern Yellow-Huai River Plain, Ministry of Agriculture, China, Jinan, China; Institut Sophia Agrobiotech, FRANCE

## Abstract

The bird cherry-oat aphid, *Rhopalosiphum padi* (L.), is a major insect pest of cereal crops in many countries. Imidacloprid has been widely used for controlling piercing-sucking insect pests worldwide, but its sublethal effects on *R*. *padi* have not been well addressed. In this study, we investigated the sublethal effects of imidacloprid on biological parameters and five enzyme activities of *R*. *padi*. The LC_10_, LC_20_, and LC_25_ of imidacloprid to adult aphids were 0.0053, 0.0329 and 0.0659 mg L^-1^, respectively. These concentrations significantly decreased pre-adult survival rate, but prolonged the development duration of 1^st^ instar nymphs, pre-oviposition period, and adult longevity. Adult oviposition period was also extended by LC_20_. The intrinsic rate of increase (*r*), net reproductive rate (*R*_0_), and finite rate (*λ*) decreased at all three concentrations, whereas mean generation time (*T*) increased. Moreover, LC_20_ and LC_25_ significantly inhibited superoxide dismutase (SOD) activity, but increased catalase (CAT) activity. Acetylcholinesterase (AChE) activity also increased at LC_20_. However, cytochrome P450 enzyme and peroxidase (POD) activity did not differ between imidacloprid treatments and the control. In conclusion, the imidacloprid concentrations tested here have negative impacts on the performance of *R*. *padi* by reducing its nymphal survival, extending the development duration of some stages, decreasing the rate of population growth, and altering enzyme activities.

## Introduction

Neonicotinoid insecticides, developed during the 1980s, have been widely applied to control piercing-sucking insect pests in various agricultural crops, with application to seeds, soil, and foliage [1–3]. They act as selective agonists of nicotinic acetylcholine receptors (nAChRs) in the central nervous system of insects, causing nervous stimulation at low to moderate concentrations, but leading to paralysis and death at high concentrations [4]. Many insect pests are effectively controlled by these insecticides [2, 3]. As systemic insecticides, they can travel through plant tissues and protect all parts of the crop [2]. However, because of their imbalanced distribution and degradation in the field [3,5,6], neonicotinoid insecticides, similar to other classes of insecticide, can not only cause direct mortality, but also result in sublethal effects on the exposed insects.

Sublethal effects have been described as effects on the physiology and behavior of an individual that has been exposed to an insecticide or toxin (the dose and/or concentration can be sublethal or lethal) [7]. Numerous studies have addressed this issue and showed that insecticides, including neonicotinoids, can cause sublethal effects on behavioral and physiological traits in arthropods, such as feeding activity, development duration, reproduction, host finding, and others [8–15]. For example, lower lethal concentrations of imidacloprid (LC_10_ and LC_30_) significantly affected the duration of phloem ingestion and decreased the developmental period of nymphs, adult longevity, and total fecundity of *Aphis gossypii* [9]. Additionally, few studies have investigated the sublethal effects of insecticides on enzymatic processes in insects [16–19]. Xiao et al. reported that acetylcholinesterase (AchE) activity decreased in both *Rhopalosiphum padi* and *Sitobion avenae* treated by LC_25_ of pirimicarb [17]. Therefore, identifying and characterizing such sublethal effects is crucial to fully understand the relationships between the exposure dose of insecticides and insect response at both the individual and population levels.

Life tables are useful tools to assess the sublethal effects of insecticides on insects at the population level [20]. The age-stage, two-sex life table approach can provide more accurate estimates of population parameters, given that this approach considers both sexes and the variable developmental rate among individuals and can properly describe the development, stage differentiation, survival, and reproduction of the population [21–23]. It has been widely used to examine the responses of various herbivore species to doses and/or concentrations of insecticides [14, 23–26]. For example, when *M*. *persicae* adults were exposed to LC_30_ of imidacloprid, the mean generation time (*T*) and gross reproductive rate (*GRR*) were significantly increased, whereas the intrinsic rate of increase (*r*) and finite rate of increase (*λ*) were reduced [24]. Thus, this method can provide a comprehensive assessment of sublethal effects of insecticides on arthropods at the population level.

The bird cherry-oat aphid, *Rhopalosiphum padi* (Hemiptera: Aphididae), a destructive pest of cereal crops worldwide, can cause direct damage by feeding on cereal plants and indirect damage by acting as vectors for the transmission of many viruses, including barley yellow dwarf virus (BYDV) [3, 27–30]. Outbreaks of this species have led to high yield losses of wheat in China [3]. Imidacloprid, the first commercially available neonicotinoid insecticide, has been applied extensively to control *R*. *padi* population in wheat fields [31]. However, its sublethal effects on *R*. *padi* have not yet been fully addressed. In this study, we investigated the sublethal effects of imidacloprid on the survival, development, fecundity, demographical parameters, and five enzyme activities of *R*. *padi*. This study aimed to determine the sublethal effects of imidacloprid on population and individual enzyme activity of *R*. *padi*; our results provide relevant information for the optimal use of imidacloprid against this pest.

## Materials and methods

### Ethics statement

No specific permits were required for the described experiments. The *R*. *padi* used in this study were originally collected from winter wheat fields at the Jinan Experimental Station of Shandong Academy of Agricultural Sciences (36.98°N, 116.98°E) and owned by the author’s institute (Institute of Plant Protection, Shandong Academy of Agricultural Sciences). None of the experiments involved any endangered or protected species.

### Insects

The *R*. *padi* adults were initially collected from winter wheat fields at the Jinan Experimental Station of Shandong Academy of Agricultural Sciences (36.98° N, 116.98° E), Shandong Province, China, in 2014 and then maintained in climatic chambers at 23±1 ^o^C, 60±10% relative humidity (RH) and a 16: 8 light: dark (L: D) photoperiod. These aphids were reared on insecticide-free wheat seedlings, which were changed weekly.

### Insecticide and enzyme kits

Imidacloprid (CAS number: 138261-41-3, 95% purity) was purchased from Shandong Sino-agri United Biotechnology Co., Ltd (Jinan, Shandong Province, China). Kits for superoxide dismutase (SOD), peroxidase (POD), catalase (CAT), acetylcholinesterase (AChE), and cytochrome P450 enzyme were purchased from Suzhou Comin Biotechnology Co., Ltd (Suzhou, Jiangsu Province, China).

### Acute toxicity of imidacloprid on *R*. *padi*

The acute toxicity of imidacloprid against *R*. *padi* adults was evaluated using the leaf-dip method [5, 32]. The stock solution of imidacloprid was prepared using analytical grade acetone, and then diluted with distilled water containing 0.5% Triton X-100 into five different concentrations: 10, 2, 0.4, 0.08 and 0.016 mg L^–1^. Leaves were cut from insecticide-free wheat plants, dipped into insecticide solutions for 30 s, and placed in the shade to air dry for 2 h. These leaves were then placed with their abaxial surface downward in a Petri dish (9.0 cm in diameter) containing 2% agar to maintain humidity. Each treatment comprised three replicates of 30 adult aphids and there were five leaves used for each replication. Distilled water containing 0.5% Triton X-100 was used as the control. The Petri dishes were placed in climatic chambers at 23±1 ^o^C, 60±10% RH, and a 16: 8 L: D photoperiod. Aphid mortalities were examined under a stereomicroscope at 24 h after exposure to imidacloprid. If the aphids were exposed to imidacloprid for longer than 24 h, the LC_50_ value obtained would be lower than that at 24h.

### Sublethal effects of imidacloprid on *R*. *padi*

LC_10_ (0.0053 mg L^–1^), LC_20_ (0.0329 mg L^–1^), and LC_25_ (0.0659 mg L^–1^) of imidacloprid were used in the life table experiment and distilled water containing 0.5% Triton X-100 was used as the control. The insecticide solutions were prepared as described in the ‘Acute toxicity of imidacloprid on *R*. *padi*’ section. The cut leaves were dipped into each imidacloprid solution and control solution for 30 s, placed in the shade to air dry for 2 h, and then put with their abaxial surface downward in a Petri dish (9.0 cm in diameter) containing 2% agar to maintain humidity. Adult aphids without exposure to any insecticide were allowed to feed on the treated leaves for 24 h. After that, the surviving adult aphids were individually transferred to new, smaller Petri dishes (3.5 cm in diameter) containing 2% agar with insecticide-free leaves. After 24 h, the adults were removed and only one nymph was left in each Petri dish. Each treatment contained 30 neonate nymphs. The survival rate, development duration, and the number of nymphs produced per aphid were recorded daily under a stereomicroscope until the death of the adult. The wheat leaves were changed every 5 days. All the experiments were conducted in climatic chambers at 23±1 ^o^C, 60±10% RH, and a 16: 8 L: D photoperiod.

### Enzyme activity determination

Adult aphids were allowed to feed on wheat leaves treated with LC_10_, LC_20_, or LC_25_ of imidacloprid and distilled water containing 0.5% Triton X-100 for 24 h. Each treatment was repeated three times. Any surviving adult aphids were collected for each replication, immediately frozen in liquid nitrogen, and stored at –80°C until use.

**Enzyme preparation**: approximately 0.1 g frozen aphids was homogenized in 1 mL ice-cold phosphate buffer (0.1 M, pH 7.5, containing 0.5% Triton X-100) and centrifuged at 8000 *g* (Eppendorf centrifuge 5417R, Germany), 4 ^o^C for 10 min. The supernatants were then used for the enzyme activity assays for SOD, POD, AChE, and P450. For CAT, approximately 0.2 g frozen aphids was homogenized in 0.2 mL normal saline and centrifuged at 8000 *g*, 4°C for 10 min. The supernatants were then used for the CAT activity assay. All optical densities (OD) were measured at different optimum wavelengths using an Emax Plus Molecular Device (Molecular Devices, Sunnyvale, California, USA).

**SOD activity**: the mixture contained 18 μL enzyme preparation, 45 μL EDTA, 100 μL xanthine, and 2 μL xanthine oxidase. The reaction was stopped by the addition of 36 μL NBT after incubation at 27 ^o^C for 30 min. The control samples contained no enzyme during the incubation. The OD was measured at 560 nm.

**POD activity:** the mixture contained 15 μL enzyme preparation, 270 μL distilled water, 520 μL sodium acetate and acetic acid solution, 30 μL hydrogen peroxide and 135 μL guaiacol solution. The reaction was stopped by the addition of 36 μL of NBT after incubation at 27 ^o^C for 30 min. The OD was measured at 30 s and 90 s at 470 nm.

**CAT activity:** the mixture contained 0.05 mL enzyme preparation, 0.1 mL hydrogen peroxide, and 0.01 mL phosphate buffer, and was incubated at 37°C for 1 min. Then, 0.1 mL reagent C and 0.01 mL reagent D were added to the mixture. The OD was measured at 240 nm.

**AChE activity:** all the reagents were added to Eppendorf tubes according to protocols of the AChE activity kit provided by Suzhou Comin Biotechnology Co., Ltd. The OD was measured at 412 nm.

**P450 activity:** the mixtures containing either 50 μL standard or sample and 50 μL enzyme conjugate was incubated at 37°C for 30 min. Then, 50 μL substrate A and 50 μL substrate B were added. Each test tube was again incubated at 37°C for 10 min in the dark. The reaction was stopped by adding 50 μL of the stop solution. The OD was measured at 450 nm within 15 min of the reaction being stopped.

### Data analysis

The mortality data for each imidacloprid treatment were corrected relative to the control group. LC_10_, LC_20_, LC_25,_ and LC_50_ were calculated by probit analysis in SPSS v18.0 software (IBM Inc., New York, USA). Data on enzyme activities were analyzed by one-way ANOVA followed by Tukey’s multiple-range test. The raw data of development and reproduction of individual insects (survival, longevity, and female daily fecundity) were analyzed using TWOSEX-MSChart [33] computer program according to the theory for the age-stage, two-sex life table [21–23]. The age- stage-specific survival rate (*s*_*xj*_, *x* is age and *j* is the stage), the age-specific survival rate (*l*_*x*_), the age-stage-specific fecundity (*f*_*xj*_), and the age-specific fecundity (*m*_*x*_) were calculated. Four demographic parameters were further obtained based on the *l*_*x*_ and *m*_*x*_. The net reproductive rate (*R*_0_) was calculated using Eq 1:

$$R_{0}=\sum_{x=0}^{\infty }l_{x}m_{x}$$

The intrinsic rate of increase (*r*) was estimated using the iterative bisection method (Eq 2):

$$\sum_{x=0}^{\infty }e^{-r(x+1)}l_{x}m_{x}=1$$

The finite rate (*λ*) was calculated using Eq 3:

$$\lambda =e^{r}$$

The mean generation time (*T*) was calculated using Eq 4:

$$T=\frac{\ln\;(R_{0})}{r}$$

The means and standard errors of life table parameters were estimated using a bootstrap procedure with a bootstrap number *m* = 100, 000 to ensure more precise estimates [34]. The paired bootstrap test was used to compare the differences in survival rate, development time, longevity, fecundity, and life table parameters among the treatments based on the confidence interval of differences [26, 35–37]. SigmaPlot 12.0 software (Systat Software Inc., San Jose, CA) was used to create the figures.

## Results

### Acute toxicity of imidacloprid on *R*. *padi*

The regression equation obtained from the concentration-mortality response bioassay at 24 h was: *Y* = 0.5609 *X* + 4.9654 (*χ*^*2*^ = 2.75, *P* = 0.432, *r* = 0.88, where *X* represents the log-transform of concentrations of imidacloprid and *Y* represents the probability value of aphid mortalities). The LC_10_, LC_20_, LC_25_, and LC_50_ of imidacloprid against *R*. *padi* adults were 0.0053 (95% confidence interval: 0.0003–0.0979 mg L^–1^), 0.0329 (0.0046–0.2373 mg L^–1^), 0.0659 (0.0125–0.3463 mg L^–1^) and 1.084 mg L^-1^ (0.3222–3.6471 mg L^–1^), respectively.

### Sublethal effects of imidacloprid on biological parameters of *R*. *padi*

The survival, development, fecundity, and life table parameters are presented in Table 1. The developmental duration of 1^st^ instar nymphs (N1) and pre-oviposition period of adults at LC_10_, LC_20_, and LC_25_ were significantly longer than those of the control. By contrast, the survival rate of pre-adult aphids was significantly lower at all these concentrations. Adult longevity was significantly lengthened by LC_20_ and LC_25_, and aphids exposed to LC_20_ also had a longer oviposition period. However, the developmental duration of 2^nd^ instar nymphs (N2) and the duration from 3^rd^ to 4^th^ instar nymphs (N3-N4) did not differ between each imidacloprid concentration and the control. The number of nymphs laid by each female adult was also not different between imidacloprid treatment and control.

**Table 1:** Effects of three concentrations of imidacloprid on biological and demographic parameters of *Rhopalosiphum padi*.

Parameter	Treatment			
	Control	LC_10_	LC_20_	LC_25_
Biological parameter				
N1 (days)	2.32±0.10b	3.76±0.25a	3.45±0.12a	3.86±0.25a
N2 (days)	1.44±0.13a	1.71±0.20a	1.61±0.11a	1.83±0.27a
N3-N4 (days)	2.52±0.16a	2.87±0.37a	2.47±0.25a	2.25±0.37a
Pre-adult survival	0.83±0.07a	0.50±0.09b	0.57±0.09b	0.40±0.09b
Pre-oviposition period (days)	6.44±0.11b	8.47±0.33a	7.65±0.26a	8.08±0.31a
Oviposition period (days)	10.64±0.75b	10.73±1.15ab	12.94±0.60a	11.92±0.75ab
Adult longevity (days)	14.56±0.89b	17.13±2.05ab	20.29±1.17a	19.84±2.07a
Fecundity (no. of nymphs per female)	37.76±3.09ab	32.65±3.52b	44.52±2.28a	39.50±2.36ab
Demographic parameter				
Net reproductive rate *(R*_*0*_*)*	31.47±3.63a	16.32±3.45b	25.23±4.23ab	15.78±3.65b
Finite rate of increase (*λ*, d^−1^)	1.37±0.02a	1.22±0.02c	1.28±0.02b	1.22±0.03bc
Intrinsic rate of increase (*r*, d^−1^)	0.32±0.01a	0.20±0.02c	0.25±0.02b	0.20±0.02bc
Mean generation time (*T*, days)	10.93±0.21b	13.99±0.56a	12.89±0.324a	13.80±0.72a

Data are expressed as mean ± standard error (SE). SE was estimated with bootstrapping (*m* = 100,000). The different lowercase letters within the same row indicate that treatments were significantly different from each other, as determined using the paired bootstrap test (*m* = 100,000) at *P* ≤0.05

The intrinsic rate of increase (*r*) and finite rate of increase (*λ*) of aphids from all exposed concentrations were significantly lower than those of the control. The aphids exposed to LC_10_ and LC_25_ also had a lower net reproductive rate (*R*_0_). However, the mean generation time (*T*) significantly increased at all the three concentrations.

**Fig 1:**
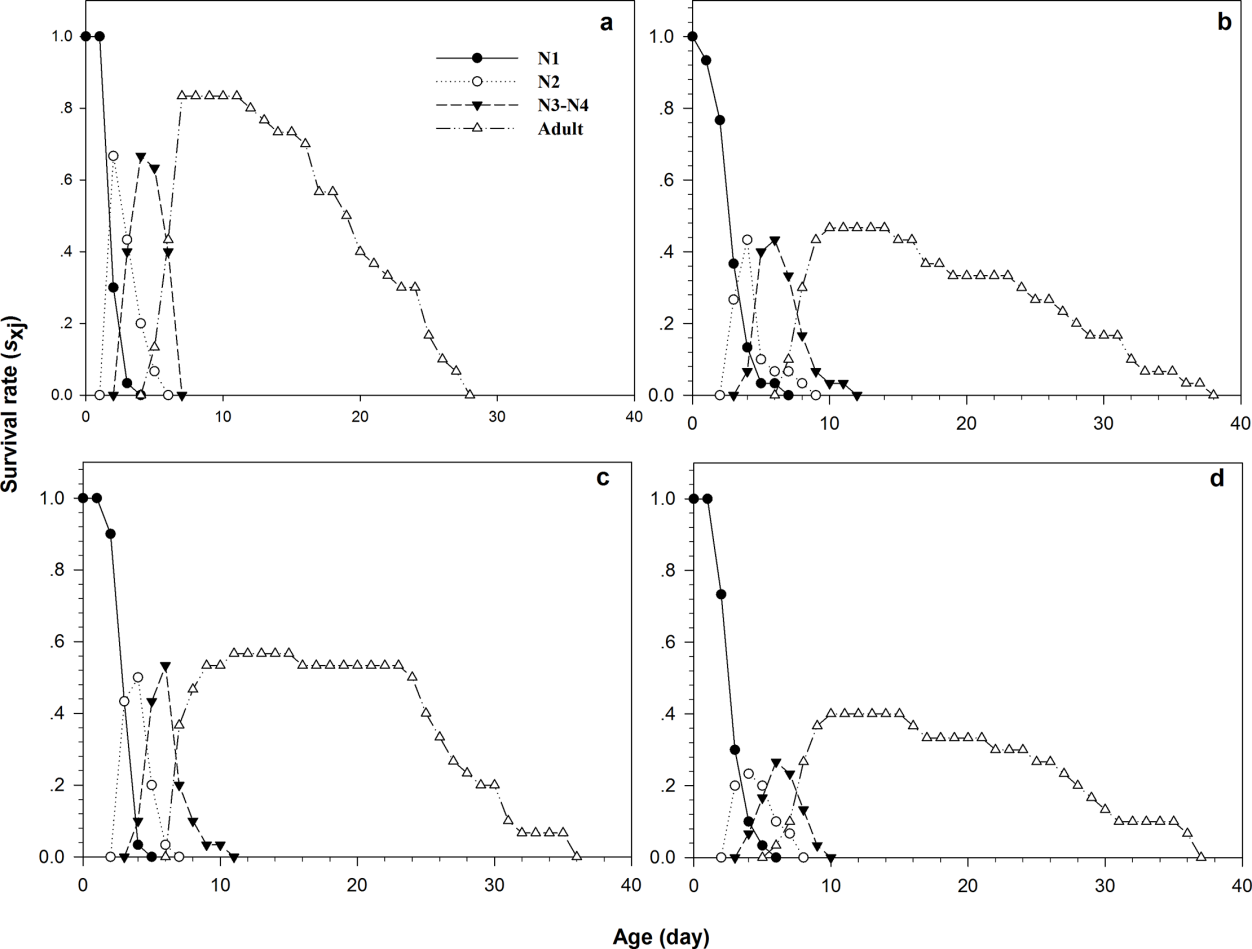
Age-stage-specific survival rates (*s*_xj_) of *Rhopalosiphum padi*, the parental females of which were exposed to wheat leaves treated with different concentrations of imidacloprid. (a) Control, (b) LC_10_, (c) LC_20_, (d) LC_25_.

The age-stage-specific survival rate (*s*_*xj*_) depicts the probability that a newborn will survive to age *x* and stage *j* (Fig 1). Given the variability in interindividual development rates, there was overlap between stages for each concentration treatment and control.

**Fig 2:**
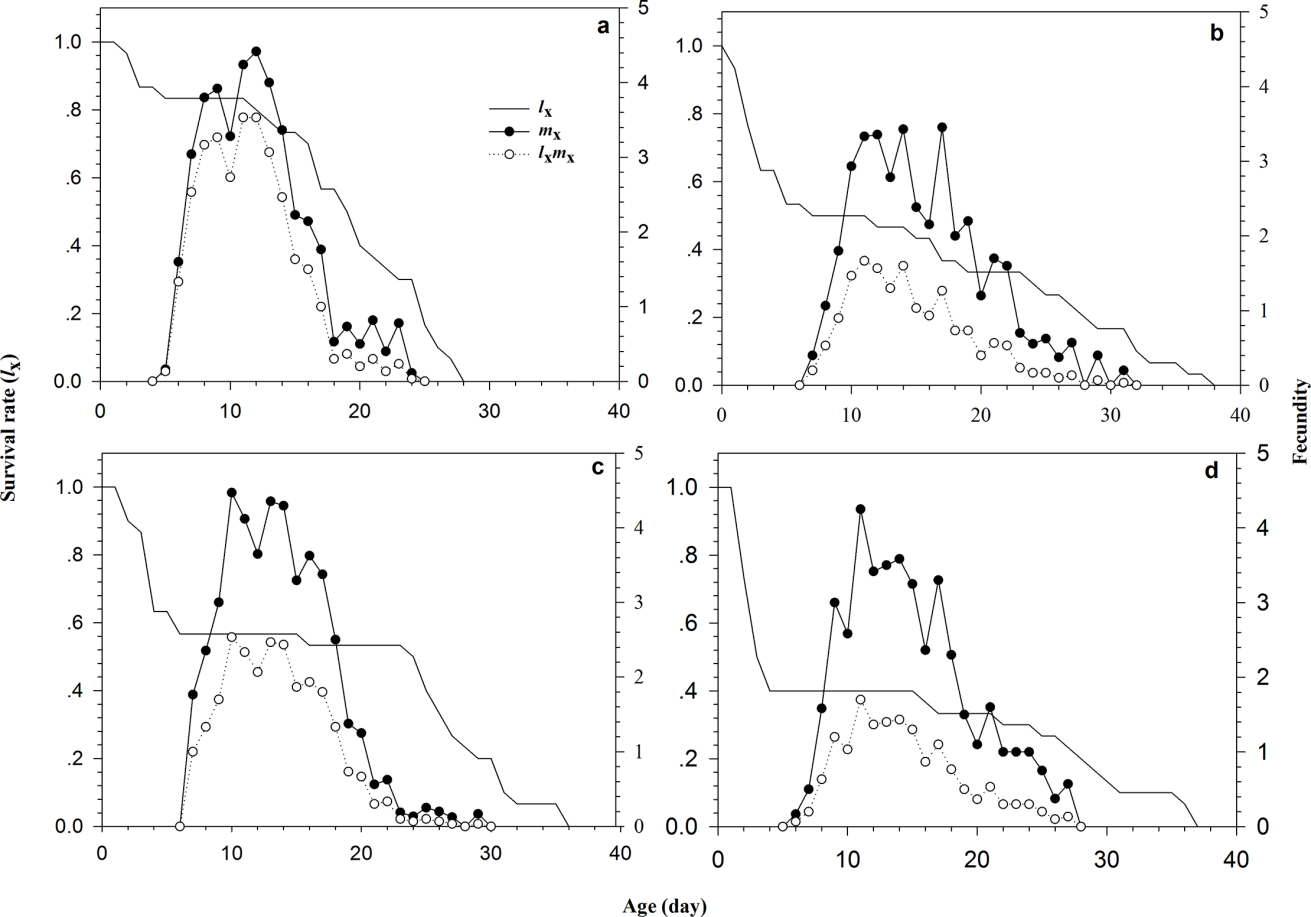
Age-specific survival rates (*l*_x_), age-specific fecundity (*m*_x_) and net maternity (*l*_x_*m*_x_) of *Rhopalosiphum padi*, the parental females of which were exposed to wheat leaves treated with different concentrations of imidacloprid. (a) Control, (b) LC_10_, (c) LC_20_, (d) LC_25_.

The age-specific survival rate (*l*_*x*_), age-specific fecundity (*m*_*x*_), and net maternity (*l*_*x*_*m*_*x*_) are shown in Fig 2. The survival rate of aphids decreased with age and the maximal survival time was 38, 36, 37, and 28 d for LC_10_, LC_20_, LC_25_, and control, respectively. In the curve *m*_*x*_, the peak fecundity occurred in the LC_10_, LC_20_, LC_25_, and control at the age of 17, 10, 11, and 12 d, respectively. The highest fecundity was 3.45, 4.47, 4.25, and 4.42 offspring, respectively. The maximal *l*_*x*_*m*_*x*_ values appeared at the age of 11, 10, 11, and 11 d, with a mean number of 1.67, 2.53, 1.70, and 3.53 offspring for the LC_10_, LC_20_, LC_25_, and control, respectively.

**Fig 3:**
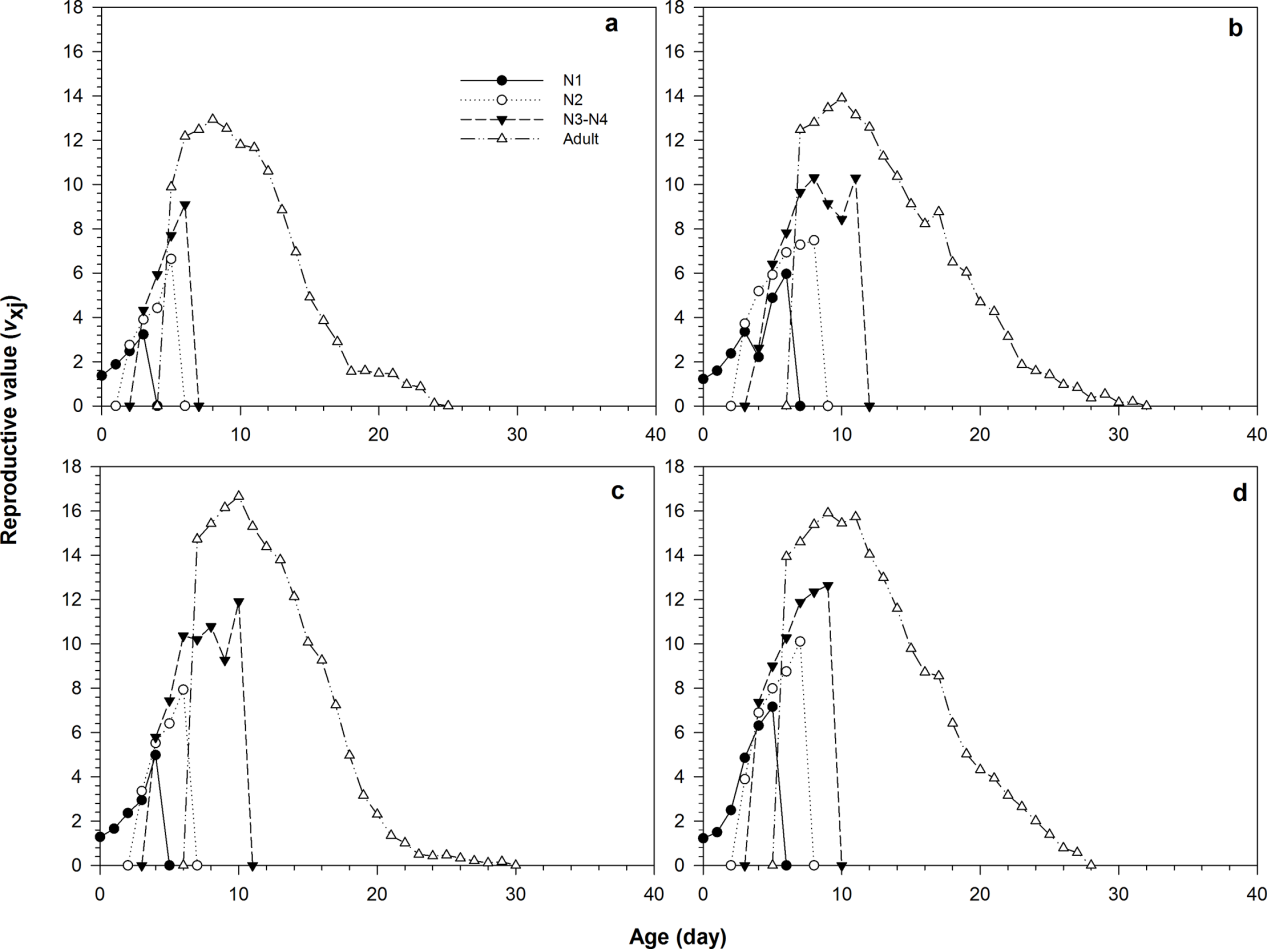
Age-stage-specific reproductive value (*v*_xj_) of *Rhopalosiphum padi*, the parental females of which were exposed to wheat leaves treated with different concentrations of imidacloprid. (a) Control, (b) LC_10_, (c) LC_20_, (d) LC_25_.

The age-stage-specific reproductive values (*v*_*xj*_, which denotes the expectation of future offspring for individuals of *R*. *padi* at age *x* and stage *j*) are shown in Fig 3. With the extension of age and stage, *v*_*xj*_ gradually increased at first and then decreased in the three treatments and control. The highest *v*_*xj*_ values occurred in the LC_10_, LC_20_, LC_25_, and control at the age of 10, 10, 9, and 8 d, respectively. The highest *v*_*xj*_ values were 13.90, 16.66, 15.91, and 12.93 offspring, respectively.

### Sublethal effects of imidacloprid on enzyme activity of *R*. *padi*

The five enzyme activities are shown in Table 2. LC_20_ and LC_25_ significantly inhibited the SOD activity of aphids, but increased CAT activity. AChE activity increased significantly at LC_20_. However, P450 and POD activities were not significantly different between the three imidacloprid treatments and control.

**Table 2:** Effects of three concentrations of imidacloprid on five enzyme activities of *Rhopalosiphum padi*.

Parameter	Treatment				Statistics	
	Control	LC_10_	LC_20_	LC_25_	*F*_3,11_	*P*
SOD (U/g)	24.51±4.60a	18.15±0.09ab	10.14±3.59b	6.91±0.93b	7.24	0.011
POD (U/g)	3444.43±1365.36a	1983.03±413.49a	3333.33±921.63a	6666.67±1272.94a	3.51	0.069
CAT (U/g)	208.62±30.11c	369.81±40.28c	801.27±71.17b	1695.00±65.24a	173.62	< 0.001
AChE (nmol/min/g)	697.88±259.85b	1742.22±394.80b	3702.22±451.22a	2014.44±260.75b	12.54	0.002
P450 (ng/100g)	59.48±10.69a	53.63±6.55a	54.36±9.08a	74.11±5.92a	1.32	0.335

Data are expressed as mean ± standard error (SE) (*n* = 3). The same lowercase letters within the same row indicate that the treatments were not significantly different from each other based on a one-way ANOVA followed by Tukey’s multiple comparisons test at *P* ≤0.05.

## Discussion

Our results showed that exposure to three concentrations of imidacloprid resulted in the duration of 1^st^ instar nymphs (N1), pre-oviposition period, and adult longevity of *R*. *padi* being significantly longer than those of the control (Table 1). These findings have been reported by previous studies on *Aphis glycines* [38], *Myzus persicae* [24], *R*. *padi* [10], and *A*. *gossypii* [39]. Zeng et al. reported that the nymphal period, pre-oviposition period, and female longevity of *M*. *persicae* were significantly prolonged when adults were exposed to LC_30_ (0.541 mg L^–1^) of imidacloprid [24]. By contrast, reduced nymphal development duration and adult longevity were observed on *A*. *gossypii* [9, 11] and *Apolygus lucorum* [15] exposed to sublethal concentrations of neonicotinoids. Koo et al. showed that the development period of *A*. *gossypii* nymphs was shorter at LC_30_ (0.82 mg L^–1^) of imidacloprid [9]. The differences in development rate of insect pests exposed to plants treated with neonicotinoid might be caused by changes in their feeding behavior. Low concentrations of imidacloprid have antifeedant effects, as determined by honeydew excretion and feeding behavior studies [9, 12, 24, 31, 39, 40]. This property can negatively affect nutrition absorption, disrupt the hormone balance, and might induce a trade-off between development and fecundity.

We also found that aphids exposed to LC_10_ and LC_25_ had a lower net reproductive rate (*R*_0_). This could be attributed to the higher mortality in nymphal stages of *R*. *padi* at these two concentrations with a mortality of 50% and 60%, respectively, compared with 17% in control (Table 1 and Fig 2). Zeng et al. reported that the pre-adult survivorship of *M*. *persicae* exposed to LC_30_ of imidacloprid was lower (70%) than that of the control (91.67%) [24]. Moreover, there was no significant difference between each imidacloprid concentration and the control in terms of the fecundity of female adult aphids. Miao et al. showed that the fertility of *Sitobion avenae* feeding on wheat plants treated with LC_10_ of imidacloprid, dinotefuran, thiacloprid, and thiamethoxam was not affected [31]. Lu et al. demonstrated that 3 h exposure to sublethal doses of imidacloprid for one generation had no discernible effect on the fecundity of either *S*. *avenae* or *R*. *padi* [18]. However, numerous studies reported that lower concentrations of imidacloprid can induce higher fecundity of exposed insects, such as *A*. *glycines* [38], *M*. *persicae* [5, 19, 24], and *A*. *gossypii* [11]. Qu et al. reported that 0.05 mg L^–1^ imidacloprid significantly increased the cumulative offspring per aphid, whereas this was significantly reduced by 0.2 mg L^–1^ [38]. This phenomenon might be related to hormesis, which is the stimulation of organism performance at low levels of exposure to a toxic agent that is normally toxic at high levels of exposure [41]. Hormesis is often implicated as the primary mechanism for the resurgence of pest populations [42, 43]. Additionally, the intrinsic rate of increase (*r*) and finite rate of increase (*λ*) of aphids from all exposed concentrations were significantly lower than those of the control (Table 1), which might indicate that the concentrations of imidacloprid tested here can suppress the population growth of *R*. *padi*. Given that fecundity did not differ between the imidacloprid treatment and control, the lower survival rate of nymphal stages in aphids from imidacloprid treatment could lead to such a difference. Zeng et al. reported that *M*. *persicae* treated with LC_30_ imidacloprid also showed lower *r* and *λ* [24]. In addition, 0.20 mg L^-1^ imidacloprid significantly reduced *r* and *λ* of *A*. *glycines* population and might suppress their population growth through a reduction in survival and reproduction activity [38]. Therefore, these three concentrations of imidacloprid could negatively impact the performance of *R*. *padi* at the population level by changing key biological parameters.

Sublethal exposure might also lead to changes in the physiological traits of insects [7]. Several studies have reported that sublethal concentrations and/or doses of insecticides altered the enzyme activities, detoxification gene expression, and carboxylesterase expression of exposed insects, such as *R*. *padi* [18], *S*. *avenae* [18], *M*. *persicae* [19], *Porcellio scaber* [44], *Megacopta cribraria* [45], *Chironomus riparius* [46], and *A*. *gossypii* [47]. Lu et al. showed that 3 h exposure to a sublethal dose of imidacloprid for one generation induced the activity of carboxylesterase (CarE) but inhibited the activity of glutathione S-transferase (GST) in *S*. *avenae* and *R*. *padi* [18]. Rix et al. showed that 0.25 and 2.5 μg L^–1^ imidacloprid significantly increased or decreased the expression of genes encoding E4-esterase and cytochrome P450-CYP6CY3 in *M*. *persicae*, with variation within and across generations [19]. In the current study, we found that lower concentrations of imidacloprid (LC_20_ or LC_25_) inhibited the SOD activity of *R*. *padi*, but increased the activities of CAT and AChE. Given that SOD activity was inhibited by imidacloprid, harmful superoxide free radicals could not be fully catalyzed into hydrogen peroxide (H_2_O_2_) and might accumulate in the body of *R*. *padi*. The increased CAT activity could help these aphids degrade some of this H_2_O_2_ and protect them from oxidative damage. Moreover, although AChE is not a molecular target of imidacloprid and has no role in the detoxification of insecticides, it is an important biochemical marker in ecotoxicology. The increased AChE activity of *R*. *padi* treated with LC_20_ indicates that an excess of acetylcholine might exist in the synaptic cleft and promote a higher activity of AChE. Another study also showed that LC_40_ of imidacloprid increased AChE activity in *M*. *cribraria* [45]. Similarly, P450 has an important role in the detoxification of xenobiotics, including pesticides, and its mediated detoxification is one of the major mechanisms of insecticide resistance [48–50]. In our experiment, the absence of effects on P450 activity demonstrates that the imidacloprid concentrations used in the life table experiment might not reach the threshold that induces the response of adult aphids. Thus, *R*. *padi* alters its enzyme activities to counter the negative effects resulting from imidacloprid.

In conclusion, our laboratory study showed that the concentrations of imidacloprid investigated negatively affected the performance of *R*. *padi* by reducing its nymphal survival, extending the development duration of some stages, suppressing population growth, and altering enzyme activities. The most important finding was that imidacloprid, even at a lower concentration (LC_10_: 0.0053 mg L^–1^), significantly reduced the survival rate of pre-adult aphids and suppressed their population growth rate. This would be helpful in controlling populations of this aphid in the field, because lower concentrations of this insecticide can effectively reduce the insect population size, reducing the ecological risks to biodiversity and beneficial insects, especially natural enemies. However, because of the complexity of field conditions, more trials should be carried out in the field to fully assess the suitability and safety of these concentrations selected in laboratory study.

## Supporting information

S1 Table Spread sheet tables presenting supplementary data. (XLSX)
